# Improved renal survival in Japanese children with IgA nephropathy

**DOI:** 10.1007/s00467-007-0726-5

**Published:** 2008-06-01

**Authors:** Nahoko Yata, Koichi Nakanishi, Yuko Shima, Hiroko Togawa, Mina Obana, Mayumi Sako, Kandai Nozu, Ryojiro Tanaka, Kazumoto Iijima, Norishige Yoshikawa

**Affiliations:** 1 Department of Pediatrics, Wakayama Medical University, 811-1 Kimiidera, Wakayama, 641-8509 Japan; 2 Department of Pediatrics, Kobe University Graduate School of Medicine, Kobe, Hyogo Japan; 3 Department of Nephrology, Hyogo Prefectural Kobe Children’s Hospital, Kobe, Hyogo Japan; 4 Department of Nephrology, National Center for Child Health and Development, Setagaya, Tokyo, Japan

**Keywords:** ACE inhibitors, Corticosteroid, Immunosuppressive drug, Mesangial proliferation, Proteinuria

## Abstract

Since the beginning of the 1990s, Japanese medical practitioners have extensively prescribed angiotensin-converting enzyme (ACE) inhibitors for children with mild IgA nephropathy (IgA-N) and steriods for those with severe IgA-N. We have performed a retrospective cohort study to clarify whether the long-term outcome has improved in Japanese children with IgA-N. Renal survival was defined as the time from onset to end-stage renal disease (ESRD). We divided the study period into two time periods based on the occurrence of the initial renal biopsy:1976–1989 and 1990–2004. Actuarial survivals were calculated by Kaplan–Meier method, and comparisons were made with the logrank test. The Cox proportional hazard model was used for multivariate analysis. Between 1976 and 2004, 500 children were diagnosed as having IgA-N in our hospitals. The actuarial renal survival from the time of apparent disease onset was 96.4% at 10 years, 84.5% at 15 years and 73.9% at 20 years. Renal survival in the 1990–2004 period was significantly better than that in 1976–1989 (*p* = 0.008), and a marked improvement in renal survival in patients with severe IgA-N was also observed (*p* = 0.0003). Multivariate analysis indicated that diagnosis year was a significant factor for ESRD-free survival independently of baseline characteristics. The results of this study show that there has been an improvement in terms of renal survival in Japanese children with IgA-N.

## Introduction

IgA nephropathy (IgA-N) is the most common primary glomerulonephritis worldwide. It was initially considered to be a benign disease with a favorable prognosis, but data from long-term follow-up studies subsequently revealed that the disease progressed to renal failure in 20–50% of adult patients [1, 2]. Although there has been a prevailing belief that the prognosis of IgA-N is more benign in children, the results of more recent studies do not support this [3]. At the beginning of the 1990s, a strict policy of treatment for IgA-N was adopted by the majority of physicians [4]; this consisted of the extensive use of angiotensin-converting enzyme inhibitors (ACEIs) as an almost universally used therapy for IgA-N, and other therapies, including corticosteroids, for more severe IgA-N [4]. This approach has also been adopted in Japan since the beginning of the 1990s, with ACEIs generally prescribed for children with mild IgA-N showing focal mesangial proliferation and steroids prescribed for children with severe IgA-N showing diffuse mesangial proliferation. Combined therapy with prednisolone, azathioprine, heparin–warfarin and dipyridamole for 2 years in patients with severe IgA-N showing diffuse mesangial proliferation was started at the Kobe University and Wakayama Medical University hospitals in 1990 [5, 6]. At present, the effect of this therapy on long-term renal survival in children with IgA-N remains unknown as there have been few studies on outcome in sufficiently large cohorts of pediatric patients [4].

In the study reported here, we investigated data from 500 children with IgA-N to determine whether the long-term outcome has improved in Japanese children with IgA-N.

## Methods

### Patients

The medical history of children under the age of 20 years who underwent routine renal biopsies at Kobe University and Wakayama Medical University hospitals between January 1976 and December 2004 were analyzed retrospectively. Clinical data and follow-up information were obtained from the medical records, and if needed, further complementary information was obtained by telephone contact with the patients, family members and or physicians.

A diagnosis of IgA-N was based on the presence of IgA as the sole or predominant immunoglobulin in the glomerular mesangium in the absence of systemic disease [7]. Diffuse or focal mesangial proliferation was defined on the basis of World Health Organization criteria [8]: diffuse mesangial proliferation was defined as more than 80% of glomeruli showing moderate or severe mesangial cell proliferation, i.e. more than three cells per peripheral mesangial area; focal mesangial proliferation was defined as less than 80% of glomeruli showing moderate or severe mesangial cell proliferation. All children with IgA-N were diagnosed by one investigator (N.Y.) using the same criteria during the entire study period. Renal biopsies were performed in children with persistent proteinuria (early morning urine protein/creatinine ratio ≥0.2g/g) with or without hematuria. Renal biopsy criteria were not changed during the entire study period.

Renal survival was defined as the period from the time of apparent disease onset to end-stage renal disease (ESRD) requiring renal replacement therapy.

Given the change of policy for treating IgA-N patients in the early 1990s, we divided the study period when the initial renal biopsy had been performed into two time periods: 1976–1989 (early period) and 1990–2004 (late period).

### Treatment

Since we started a strict policy of treatment for IgA-N at the beginning of the 1990s, we investigated the initial treatment profile of children with IgA-N. After a pilot study period, we started the first randomized controlled trial (RCT) by the Japanese Pediatric IgA Nephropathy Treatment Study Group (JPIGANTS) for treatment of children with severe IgA-N showing diffuse mesangial proliferation in 1990 [5]. We started the second RCT by the JPIGANTS for treatment of children with severe IgA-N in 1994 [6]. We started the third RCT by the JPIGANTS for treatment of children with severe IgA-N in 2001. The details of each treatment are shown in Table 1.

**Table 1:** Treatment in three randomized controlled trials for childhood IgA nephropathy with diffuse mesangial proliferation

Period	1990–1993 (first RCT)	1994–1998 (second RCT)	2001–present (third RCT)
Group 1	Prednisolone	Prednisolone (as in group 1 in the first period)	Prednisolone
	2 mg/kg/day, 4 weeks		2 mg/kg/day, 4 weeks
	2 mg/kg/2 days, 4 weeks		2 mg/kg/2 days, 4 weeks
	1.5 mg/kg/2 days, 4 weeks		1.5 mg/kg/2 days, 4 weeks
	1 mg/kg/2 days, 21 months		1 mg/kg/2 days, 9 months
			0.5 mg/kg/2 days, 12 months
	Azathioprine	Azathioprine (as in group 1 in the first period)	No azathioprine
	2 mg/kg/day, 24 months		
	Dipyridamole	Dipyridamole (as in group 1 in the first period)	Dipyridamole
	5 mg/kg/day, 24 months		6–7 mg/kg/day, 24 months
	Heparin	No heparin	
	APTT 60 s, 28 days		
	Warfarin	Warfarin	Warfarin
	TT 30–50%, 23 months	TT 30–50%, 24 months	TT 20–50%, 24 months
			Mizoribine
			4 mg/kg/day, 24 months
Group 2	Heparin-Warfarin		
	Dipyridamole (as in group 1)		
		Prednisolone (as in group 1)	Prednisolone (as in group 1)
			Mizoribine (as in group 1)

TT, Thrombotest; APTT, activated partial thromboplastin time; RCT, randomized controlled trial

For the treatment of children with mild IgA-N showing focal mesangial proliferation we started a RCT by the JPIGANTS in 1990. During this trial (1990–1993) about half of the children with mild IgA-N in our hospitals received Sairei-to, a Chinese herb, for 24 months, and the other half received no medication. We began to use ACEIs for treatment of mild IgA-N with focal mesangial proliferation as the first choice from the beginning of 2000.

### Statistical analyses

The results were analyzed with SAS ver. 9.1.2 (SAS Institute Japan Ltd., Tokyo, Japan). The associations between categorical variables were examined using the Fisher’s exact test. Continuous characteristics of the groups were compared with the Mann-Whitney *U*-test. Actuarial survival curves were calculated according to the Kaplan-Meier method [9], and comparisons were made with the logrank test [10]. Univariate and multivariate analyses were performed to assess the difference between the two periods in terms of renal survival. For multivariate analysis, we used the Cox proportional hazard model [11]. A two-tailed *p* value of less than 0.05 was taken as the level of significance.

## Results

### Patients

Between 1976 and 2004, 1759 children underwent a first renal biopsy examination at the Kobe University and Wakayama Medical University hospitals. Among these, 500 Japanese children (28.4%; 279 boys and 221 girls) were diagnosed as having IgA-N: 219 in 1976–1989 and 281 in 1990–2004. There was no evident change in the number of patients per year between the early (1976–1989) and late (1990–2004) periods. The median patient age at diagnosis was 10.9 years (range 2.5–19.6 years), and the median follow-up period for the patients overall was 5.9 years (range 1.3–20.5 years).

The baseline characteristics of children with IgA-N are shown in Table 2. There were significant differences in some characteristics between the two periods. Age at diagnosis had a tendency to be higher in the late group, while the ratio of asymptomatic proteinuria and hematuria at initial presentation was significantly higher in the late group. The ratio of heavy proteinuria (≥ 1 g/m^2^ per day) at diagnosis was significantly higher in the early period, but the ratio of patients showing diffuse mesangial proliferation was higher in the late period. Based on these data, we concluded that there was no evident difference in disease severity between the two periods.

**Table 2:** Baseline characteristics

Characteristic	Number of patients (%)			*p*
	Total (*n* = 500)	1976–1989 (*n* = 219)	1990–2004 (*n* = 281)	
Sex (M/F)	279/221	132/87	147/134	0.08
Age at diagnosis, year, median [range]	10.9 [2.5–19.6]	10.1 [3.4–16.8]	11.6 [2.5–19.6]	< 0.001
Initial presentation				
Asymptomatic proteinuria and hematuria	384 (76.8%)	150 (68.5%)	234 (83.3%)	< 0.001
Macroscopic hematuria	93 (18.6%)	60 (27.4%)	33 (11.7%)	< 0.001
Edema	23 (4.6%)	9 (4.1%)	14 (5.0%)	0.67
Proteinuria at diagnosis (g/m^2^/day)				
<1	361 (72.2%)	146 (66.7%)	215 (76.5%)	0.008
≥1	139 (27.8%)	73 (33.3%)	66 (23 .5%)	
Estimate creatinine clearance at diagnosis (ml/min per 1.73 m^2^)				
<60	13 (2.6%)	3 (1.4%)	10 (3.6%)	0.16
≥60	487 (97.4%)	216 (98.6%)	271 (96.4%)	
Renal biopsy at diagnosis				
Diffuse mesangial proliferation	171 (34.2%)	63 (28.8%)	108 (38.4%)	0.03
Focal mesangial proliferation	329 (65.8%)	156 (71.2%)	173 (61.6%)	

### Treatment

The initial treatment for IgA-N showing focal mesangial proliferation or diffuse mesangial proliferation is given in detail in Table 3. There was a clear change of treatment for IgA-N between the early and late periods. This is particularly true in terms of ACEI use for focal mesangial proliferation and combined therapies for diffuse mesangial proliferation, both of which increased dramatically during the late period.

**Table 3:** Change of initial treatment for IgA nephropathy in Japanese children

Treatment	Focal mesangial proliferation		Diffuse mesangial proliferation	
	1976–1989 (*n* = 156)	1990–2004 (*n* = 173)	1976–1989 (*n* = 63)	1990–2004 (*n* = 108)
No treatment	96 (61.6%)	23 (13.2%)	19 (30.2%)	1 (0.9%)
Antiplatelet and/or anticoagulant	35 (22.4%)	2 (1.2%)	14 (22.2%)	7 (6.5%)
Prednisolone, (± antiplatelet and/or anticoagulant)	4 (2.6%)	6 (3.5%)	7 (11.1%)	25 (23.1%)
Prednisolone + immunosuppressant, (± antiplatelet and/or anticoagulant)	7 (4.5%)	8 (4.6%)	19 (30.2%)	74 (68.5%)
Chinese herb (Sairei-to)	14 (9.0%)	46 (26.5%)	4 (6.3%)	0 (0.0%)
ACEI and/or ARB	0 (0.0%)	88 (50.9%)	0 (0.0%)	1 (0.9%)

ACEI, Angiotensin-converting enzyme inhibitor; ARB, angiotensin II receptor blocker

### Renal survival

**Fig. 1:**
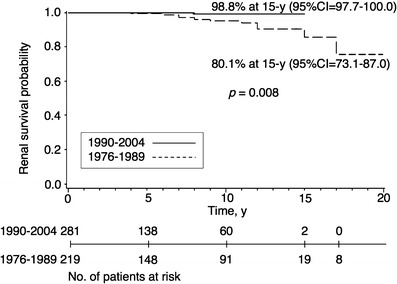
Kaplan–Meier plot of renal survival stratified by the initial biopsy year for children with IgA nephropathy. *95% CI* 95% Confidence interval

Among a total of 500 patients who had IgA-N therapy, the actuarial renal survival from the time of apparent disease onset was 96.4% at 10 years, 84.5% at 15 years and 73.9% at 20 years (Table 4). As shown in Fig. 1 and Table 4, in children diagnosed as having IgA-N in 1976–1989, the actuarial renal survival from the time of apparent disease onset was 94.0% at 10 years, 80.1% at 15 years and 70.1% at 20 years. In children diagnosed as having IgA-N in 1990–2004, the actuarial renal survival from the time of apparent disease onset was 98.8% at 10 years, 98.8% at 15 years (*p* = 0.008; Fig. 1, Table 4).

**Table 4:** Actuarial renal survival analysis of 500 children with IgA nephropathy

	Initial renal biopsy year	Number of patients	10-year renal survival	15-year renal survival	20-year renal survival	*p* ^a^
Total	1976–2004	500	96.4%	84.5%	73.9%	
Total	1976–1989	219	94.0%	80.1%	70.1%	0.008
	1990–2004	281	98.8%	98.8%	–	
Diffuse mesangial proliferation	1976–1989	63	78.5%	68.6%^b^	–	0.0003
	1990–2004	108	97.8%	97.8%^b^	–	
Focal mesangial proliferation	1976–1989	156	100.0%	97.7%	–	0.5
	1990–2004	173	100.0%	100.0%	–	

^a^*p* values on the logrank test

^b^The 13-year survival

**Fig. 2:**
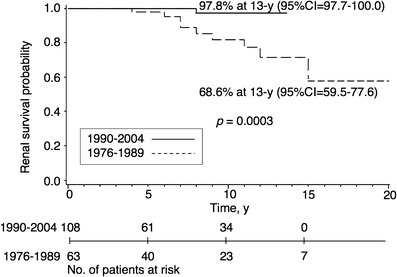
Kaplan-Meier plot of renal survival stratified by the initial biopsy year for children with severe IgA nephropathy showing diffuse mesangial proliferation

**Fig. 3:**
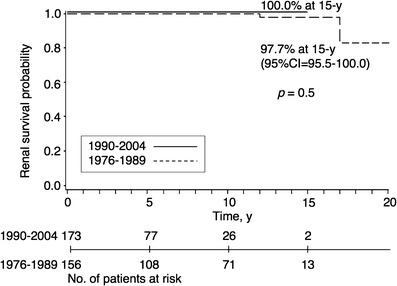
Kaplan-Meier plot of renal survival stratified by the initial biopsy year for children with mild IgA nephropathy showing focal mesangial proliferation

Figures 2 and 3 show the long-term outcome for children in the two different mesangial proliferation groups. For children with severe IgA-N showing diffuse mesangial proliferation, both the 10- and 13-year renal survivals were 97.8% when the diagnosis was made in the period 1990–2004; when the diagnosis was made in the period 1976–1989, renal survivals were 78.5% and 68.6%, respectively (*p* = 0.0003; Fig. 2, Table 4). For children with mild IgA-N showing focal mesangial proliferation, both the 10- and 15-year renal survivals were 100.0% when diagnosis was made in 1990–2004, compared with 100.0% and 97.7%, respectively, in 1976–1989 (*p* = 0.5; Fig. 3, Table 4). Although we observed better renal survival in patients diagnosed in the period 1990–2004 than in the period 1976–1989 for children with IgA-N showing focal mesangial proliferation, the difference did not reach statistical significance. The children with IgA-N showing diffuse mesangial proliferation in 1990–2004 had excellent long-term renal survival.

Table 5 shows the results of the univariate analysis using the logrank test and the multivariate analysis using the Cox proportional hazard model of prognostic factors for ESRD-free survival. Mesangial proliferation degree (focal or diffuse), proteinuria at diagnosis (<1 or ≥1 g/m^2^ per day), estimate of creatinine clearance at diagnosis (≥60 or <60 ml/min per 1.73 m^2^) and initial renal biopsy (diagnosis) year (1976–1989 or 1990–2004) were included as factors for analyses. Mesangial proliferation degree and initial renal biopsy year were significant in both the univariate and the multivariate analysis. For children with IgA-N, the most influential prognostic variable was mesangial proliferation degree. Proteinuria at diagnosis was significant in the univariate but not in the multivariate analysis. These results from the multivariate analysis showed that initial renal biopsy year was a significant factor for ESRD-free survival independently of mesangial proliferation degree, proteinuria at diagnosis and estimate of creatinine clearance at diagnosis (hazard ratio = 0.08, 95% CI 0.004–0.43; Table 5).

**Table 5:** Univariate and multivariate analysis of the prognostic value of factors for end-stage renal disease-free survival

Factor	Univariate			Multivariate		
	HR	95% CI	*p*	HR	95% CI	*p*
Mesangial proliferation focal; diffuse	10.92	2.80–72.46	<0.001	10.27	2.42–70.75	0.001
Proteinuria at diagnosis <1; ≥1 (g/m^2^/day)	5.27	1.65–19.77	0.01	2.14	0.57–8.78	0.26
CCl at diagnosis ≥ 60; < 60 (ml/min per m^2^)	14.12	2.11–57.41	0.01	5.58	0.74–30.22	0.09
Initial renal biopsy year 1976–1989; 1990–2004	0.14	0.01–0.74	0.02	0.08	0.004–0.43	0.002

CI, Confidence interval; HR, hazard ratio; CCl, creatinine clearance

## Discussion

Although this was a retrospective study, the data seem to provide unique and valuable information about IgA-N in children for several reasons. First, the study subjects were a complete non-selected cohort of patients with IgA-N from among all children who underwent first renal biopsy examinations at Kobe University and Wakayama Medical University hospitals between 1976 and 2004. Five hundred children with IgA-N participated in our study, which to our knowledge is the largest series used to investigate the outcome of IgA-N in children to date. Furthermore, these patients were diagnosed histologically by a single investigator (N.Y.) based on the same criteria during the whole study period. Renal biopsy criteria were also unchanged during the whole study period. Therefore, the cohort in the present study is considered to have been rigidly homogeneous in terms of the analysis of the disease outcome.

One of the conditions that facilitated our study was the school screening program, which was started by the Japanese government in 1974. In Japan, all children between the ages of 6 and 18 years are screened annually, and those found to have urinary abnormalities are referred for further investigation. Thus, in general, treatment for IgA-N is started early in the course of disease because the duration of disease before treatment is short as a result of this school screening program [12]. Therefore, in this study we were able to observe the disease courses of patients during the initial renal biopsy period, 1976–2004, who underwent initial treatments for IgA-N at a relatively homogeneous stage of the disease. Accumulated experience indicates that long-term corticosteroid and/or immunosuppressive treatment during the severely progressive stage of the disease does not confer any benefit in adult patients [2]. In contrast, it is known that centers in countries with active urine screening programs are more likely to diagnose mild disease with a good prognosis, thus favorably influencing the overall outcome of the cohort [13].

Another favorable parameter of this study was the enforcement of consecutive systemic nationwide clinical trials of IgA-N. After a pilot study period, the first RCT for treatment of children with severe IgA-N showing diffuse mesangial proliferation was started by the JPIGANTS in 1990 [5]. To date, two RCTs of treatment for children with severe IgA-N showing diffuse mesangial proliferation by the JPIGANTS have been completed [5, 6], and the third RCT of treatment for children with severe IgA-N showing diffuse mesangial proliferation is currently being conducted by the same group. For the treatment of children with mild IgA-N and focal mesangial proliferation, a RCT was also started by the JPIGANTS in 1990. After completion of the RCT, we have conducted prospective clinical trials of treatment for children with mild IgA-N and focal mesangial proliferation. These prospective trials have enabled for us to clarify the optimum treatment for IgA-N and to collect precise information on patients with IgA-N.

Because of the variable rate of progression to renal failure and the probable multifactorial pathogenesis of IgA-N, it is desirable to evaluate the effectiveness of any treatment by a prospective controlled trial. However, although the ultimate endpoint in any clinical trial of progressive IgA-N is the development of chronic renal insufficiency, most pediatric patients do not develop it during the study period [14]. Therefore, data from a properly analyzed long-term retrospective study may also be important to evaluate disease outcome and treatment effectiveness [4, 9–11]. Retrospective studies with accurate statistical evaluation using life-time analysis and multivariate survivorship analysis according to the Cox regression model are thought to work effectively together with well-designed RCTs.

The primary purpose of this study was to clarify whether long-term outcome was improved in Japanese children with IgA-N retrospectively using adequate statistical methods. Although it seemed that there were no clinically significant differences in the baseline characteristics of the groups, there were some statistically significant differences in the baseline characteristics of the groups between the two periods when initial renal biopsies were performed. These different baseline characteristics may influence the final outcomes reported in this study. To compensate for this limitation, we used a multivariate survivorship analysis according to the Cox regression model. Since we have to adjust for differences of baseline characteristics, which were thought to be important prognostic factors [15], mesangial proliferation degree, proteinuria at diagnosis, estimate of creatinine clearance at diagnosis and the initial renal biopsy (diagnosis) year were included as factors for analyses. This multivariate analysis indicated that the initial renal biopsy year was a significant factor for ESRD-free survival after adjustment of these baseline characteristics. These data support an improvement of renal survival in Japanese children with IgA-N.

There was a notable lack of the usual dominance of males over females in our study and, in addition, there was a difference between the 1976–1989 and the 1990–2004 periods, with more females being recruited in the second period. Although this difference was not statistically significant (*p* = 0.08), we decided that it be worthwhile discussing possible reasons for the differences in gender representation between the two periods. Therefore, we analyzed males and females separately. However, there was no gender-based factor in the results (data not shown).

At the beginning of the 1990s, a strict policy of treatment was adopted by the majority of physicians in Japan. It is likely that therapeutic intervention early after the onset of clinically apparent disease will provide the best opportunity for improving the outcome of patients with IgA-N, thus reducing the number of patients who develop ESRD [5, 6]. This study showed a clear change in the treatment of IgA-N between the early and late groups (Table 3). In particular, the use of ACEIs for focal mesangial proliferation and combination therapies for diffuse mesangial proliferation increased dramatically in the late group.

Corticosteroids have been widely used to treat moderate to severe IgA-N, particularly in pediatric patients. Some evidence has been obtained recently pertaining to the role of corticosteroids in the treatment of IgA-N [2, 13, 14, 16–18]. With regard to children and based on the results of two multicenter RCTs, we reported previously that the treatment of childhood IgA-N with diffuse mesangial proliferation using prednisolone, azathioprine, warfarin and dipyridamole for 2 years early in the course of disease reduced the severity of immunologic renal injury and prevented any increase in the percentage of sclerosed glomeruli [5, 6]. In contrast, in the first RCT, heparin–warfarin and dipyridamole treatments for 2 years did not reduce urinary protein excretion, serum IgA concentration and mesangial IgA deposition, and they did not prevent any increase of sclerosed glomeruli [5]. The results of the second RCT showed that the treatment of children with severe IgA-N using prednisolone alone for 2 years reduced the severity of immunologic renal injury, but did not prevent any further increase of glomerular sclerosis [6]. Therefore, treatment with the combination therapy using prednisolone and immunosuppressant, such as azathioprine, may be better than the prednisolone monotherapy for patients with severe IgA-N.

We found an improvement of long-term renal survival in Japanese children with IgA-N (Table 4, Figs. 1 and 2). Renal survival in 1990–2004 was significantly better than in 1976–1989 (Fig. 1), and a marked improvement of renal survival in IgA-N showing diffuse mesangial proliferation was observed over time (Fig. 2). This improvement may be related to the 2-year therapy, including corticosteroids, for all patients with IgA-N showing diffuse mesangial proliferation as a treatment policy, although in principle the effectiveness of any treatment can only be evaluated properly by a controlled trial. It is conceivable that the outcome in the period 1976–1989 may have reflected the natural course of disease, whereas that in 1990–2004 may have reflected modification of the disease course by the treatments.

The median follow-up period of the patients overall was 5.9 years (range 1.3–20.5 years). Although this is a considerably long period, it may not be sufficiently long for the follow-up of IgA-N, which has a slow progressive course. Since the elapsed time from apparent disease onset to ESRD was used to evaluate the disease outcome, chronic renal insufficiency before ESRD was not considered in our study. Thus, we do not know whether the observed improvement of outcome means a complete cure of the disease or merely a delay of the disease course. Further follow-up of the cohort for a long period is therefore important.

One aspect which we should consider in our study is the possibility that at least some of the differences in outcome between the early and late groups may be due to general changes in care that have occurred over time. Such changes are frequently not recognized, such as a gradually greater awareness of managing marginally raised blood pressure, among others.

In conclusion, this study has demonstrated a substantial improvement of long-term renal survival in Japanese children with IgA-N. On the basis of the results of this study we were unable to provide the reason for this improved outcome directly; however, it is likely that adequate management using a strict policy of treatment may have been responsible.

## References

[1] Donadio JV, Grande JP (2002). IgA nephropathy. N Engl J Med.

[2] Alexopoulos E (2004). Treatment of primary IgA nephropathy. Kidney Int.

[3] Yoshikawa N, Tanaka R, Iijima K (2001). Pathophysiology and treatment of childhood IgA nephropathy. Pediatr Nephrol.

[4] D’Amico G (2000). Natural History of Idiopathic IgA nephropathy: role of clinical and histological prognostic factors. Am J Kidney Dis.

[5] Yoshikawa N, Ito H, Sakai T, Takekoshi Y, Honda M, Awazu M, Ito K, Iitaka K, Koitabashi Y, Yamaoka K, Nakagawa K, Nakamura N, Matsuyama S, Seino Y, Takeda N, Hattori S, Ninomiya M (1999). A controlled trial of combined therapy for newly diagnosed severe childhood IgA nephropathy. J Am Soc Nephrol.

[6] the Japanese Pediatric IgA Nephropathy Treatment Study Group (2006). Steroid treatment for severe childhood IgA nephropathy: a randomized controlled trial. Clin J Am Soc Nephrol.

[7] Yoshikawa N, Ito H, Yoshiara S, Nakahara C, Yoshiya K, Hasegawa O, Matsuo T (1987). Clinical course of IgA nephropathy in children. J Pediatr.

[8] Churg J, Bernstein J, Glassock RJ (1995) Renal disease: classification and atlas of glomerular diseases, 2nd edn. Igaku-shoin Medical Publ, Tokyo

[9] Kaplan EL, Meier P (1958). Nonparametric estimation from incomplete observations. J Am Stat Assoc.

[10] Mantel N (1966). Evaluation of survival data and two new rank order statistics arising in its consideration. Cancer Chemother Rep.

[11] Cox DR, Oakes D (1984) Analysis of survival data. Chapman & Hall, London

[12] Nakanishi K, Honda M, Yoshikawa N (2004) Pediatric nephrology around the world: Japan. In: Avner ED, Harmon WE, Niaudet P (eds) Pediatric nephrology, 5th edn. Lippincott Williams & Wilkins, Philadelphia, pp 1491–1493

[13] Barratt J, Feehally J (2006). Treatment of IgA nephropathy. Kidney Int.

[14] Wyatt RJ, Hogg RJ (2001). Evidence-based assessment of treatment options for children with IgA nephropathies. Pediatr Nephrol.

[15] Yoshikawa N, Ito H, Nakamura H (1992). Prognostic indicator in childhood IgA Nephropathy. Nephron.

[16] Samuels JA, Strippoli GF, Craig JC, Schena FP, Molony DA (2004). Immunosuppressive treatments for immunoglobulin A nephropathy: a meta-analysisof randomized controlled trials. Nephrology (Carlton).

[17] Pozzi C, Bolasco PG, Fogazzi GB, Andrulli S, Altieri P, Ponticelli C, Locatelli F (1999). Corticosteroids in IgA nephropathy: a randomized controlled trial. Lancet.

[18] Pozzi C, Andrulli S, Del Vecchio L, Melis P, Fogazzi GB, Altieri P, Ponticelli C, Locatelli F (2004). Corticosteroid effectiveness in IgA nephropathy: long-term results of a randomized, controlled trial. J Am Soc Nephrol.

